# Description of a new species of *Mediotipula* from Albania, with consideration of the eastern Mediterranean as a diversity hotspot (Diptera, Tipulidae)

**DOI:** 10.3897/zookeys.792.25683

**Published:** 2018-10-23

**Authors:** Lujza Keresztes, Jesús Martínez Menéndez, Luis Martin, Edina Török, Levente-Péter Kolcsár

**Affiliations:** 1 Hungarian Department of Biology and Ecology, Centre of Systems Biology, Biodiversity and Bioresources, Faculty of Biology and Geology, University of Babeș-Bolyai Cluj-Napoca, Clinicilor 5-7, Romania University of Babeș-Bolyai Cluj-Napoca Romania; 2 Department Zoología, Antropología Física & Genética, Faculty of Biology, University of Santiago de Compostela, R/Lope Gómez de Marzoa, s/n. Campus Vida. 15782 Santiago de Compostela, Spain University of Santiago de Compostela Santiago de Compostela Spain; 3 Romanian Academy Institute of Biology, Splaiul Independenţei 296, 060031 Bucureşti, Romania Romanian Academy Institute of Biology Bucureşti Romania

**Keywords:** Craneflies, *
Mediotipula
*, new species, morphological diversity, Mediterranean hotspots, the Balkans

## Abstract

A new species of the TipulasubgenusMediotipula is described from the south-eastern part of Albania, south-eastern Europe. Morphologically, the new species is most similar to T. (M.) stigmatella Schummel, 1833, but differs mainly with respect to males, having a distinctly shaped posterior margin of tergite 9–10, a widened outer gonostylus and a series of details of the inner gonostylus (anterior end of the anterior arm, shape of the posterior arm), as well as having more bulbous and rounded hypogynal valves in the females. Further morphological differences of the male terminalia between allopatric populations of T. (M.) stigmatella in the Carpathians and Balkans, south-eastern Europe, are discussed.

## Introduction

The Mediterranean region of Europe is one of the most species-rich biomes in the world with a high level of endemism shaped by tectonic shifts (Hewitt 2011, Oosterbroek and Arntzen 1992, Markova et al. 2010), Pleistocene climatic oscillations (Previšić et al. 2014), environmental heterogeneity (Triantis et al. 2005, Pinkert et al. 2017), and/or natural selection (Kraitsek et al. 2008, Rodriguez-Ramirez et al. 2017). Biodiversity hotspots from such areas as Iberia, the Apennines, the Balkans (south-eastern Europe), the Mediterranean islands, Northwest Africa, western Turkey and the Caucasus (at the border of Europe and Asia) all have a decisive role in the glacial survival of numerous endemic or range-restricted taxa (De Jong 1998). Furthermore, they also provided a continuous supply of European biodiversity during several postglacial periods (Zachos and Habel 2011).

The western Palaearctic Tipula (Mediotipula) Pierre, 1924 is a small subgenus of only eleven species of moderately sized craneflies (Oosterbroek 2018). According to Savchenko (1966, 1979, 1983), Theowald (1978) and De Jong (1995), Mediotipula is the sister group of the subgenus Savtshenkia Alexander, 1965. However, all members of *Mediotipula* are unique among *Tipula*, by having a relatively small discoidal cell, while almost all the other *Tipula* have a relatively large discoidal cell (length-width ratio of approximately two or more). Other synapomorphies include the entirely fused, plate-like posterior apodemes of the sperm pump, in addition with a laterally compressed projection on the posterolateral corner of the gonocoxite in males (De Jong 1995). Additionally, females have the hypogynal valves fused for approximately one-half to two thirds of the length, in comparison with the totally separated (up to their bases) hypogynal valves in females of other *Tipula*. Sternite VIII of the females has a conspicuous sclerotisation of the ventral wall of the genital chamber near the openings of the gonopore, together with a usually less distinct and smaller posteroventral sclerite. Sternite IX is present as a slender and well-sclerotised structure, sometimes with a membranous medial part (De Jong 1995).

The majority of *Mediotipula* taxa have an isolated distribution in the western Palearctic area, showing high levels of endemism corresponding with the major biodiversity hotspots around the Mediterranean Sea while four species have a distribution area that is limited to the Iberian Peninsula (Oosterbroek 2018). The Balkans contain only three species with their ranges also covering Central Europe or the Caucasus. Only one species, *T.* (*M.*) *mikiana* Bergroth, 1888 has an exclusively extra-Mediterranean distribution, and only three species are range-restricted, one in Anatolia, (Asian Turkey) (*T.* (*M.*) *anatoliensis* Theowald, 1978), one in the Caucasus (*T.* (*M.*) *caucasiensis* Theowald, 1978) and one in Algeria (North Africa), (*T.* (*M.*) *fulvogrisea* Pierre, 1924) (Oosterbroek 2018).

Species of *Mediotipula* are distributed mostly in colline- to montane-subalpine ecosystems with a high level of humidity, often in very steep oak woods, and are rarely associated with open woods and hedges exposed to the sun (pers. obs., but see also Dufour 1986 and De Jong 1995). Larvae are considered to be bryobionts, and are frequently collected under moist moss in/or along brooks, or banks of rivers and even under dry moss in the case of T. (M.) stigmatella (Savchenko 1966, Theowald 1967, Höchstetter 1963).

A comprehensive phylogenetic analyses of the species which belong to *Mediotipula* was published by De Jong (1995), who compared and analyzed 24 morphological characters of both sexes (with the exception of the female of *T.* (*M.*) *fulvogrisea*), using parsimony. According to his phylogeny, only the previous *T.* (*M.*) *brolemanni* group (sensu Theowald, 1978) proved to be monophyletic, and together with the species *T.* (*M.*) *siebkei* and *T.* (*M.*) *caucasiensis* constituted a well-defined group within the subgenus *Mediotipula*.

In this paper we provide a morphological description of a new species and discuss its relationship with the similar *T.* (*M.*) *stigmatella*, based upon morphological features of the male and female terminalia. The morphological differences of male genital structures of different populations of *T.* (*M.*) *stigmatella* from the Carpathians and the Balkans are also discussed.

## Materials and methods

**Figure 1.**
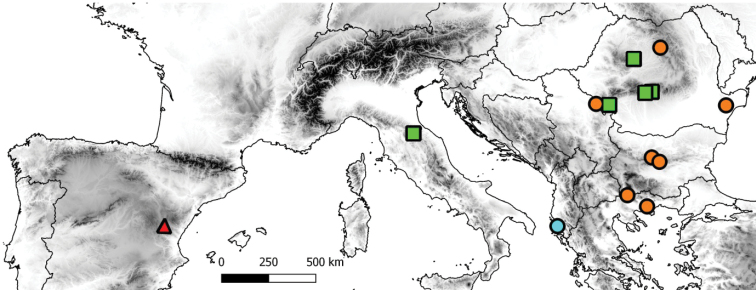
Sampling localities of different *Mediotipula* species investigated in this study. Tipula (Mediotipula) cataloniensis Theowald, 1978 (red triangle), T. (M.) stigmatella Schummel, 1833 (orange dots), T. (M.) gjipeensis sp. n. (blue dot), *T.* (*M.*) *siebkei* Zetterstedt, 1852 (green square).

Adults were collected using sweep nets and then stored in 96% ethanol or dry-pinned. Altogether, 54 male and four female individuals of *Mediotipula* belonging to four species were examined (see Table 1). All collection data are available in the TransDiptera Online Database (Kolcsár et al. 2018). Male and female genitalia were examined after being cleared in 10% KOH. Layer photos were taken using an Olympus SZ61 stereomicroscope equipped with a Canon 650D camera and LM Digital SLR Adapter; photos were combined using the Zerene Stacker software. Male and female genitalia terminology is in accordance with De Jong (1995). All specimens used in the present study were collected by the authors and deposited in the Diptera Collection of the Faculty of Biology and Geology, University Babes-Bolyai, Cluj Napoca, Romania (DCFBG) (Figure 1, Table 1).

**Table 1.** Material of the subgenus Mediotipula used in our study with localities (BG-Bulgaria, FYM-Macedonia, GR-Greece, IT-Italy, ES-Spain), geographic coordinates (given in decimal degrees), number of individuals found at each collection site, number of individuals used in the present study, date of the collection, and name of collector (leg.).

Species	Specimen	Location	Collection date	Latitude	Longitude	Collector(s)
T. (M.) cataloniensis Theowald, 1978	2♂	ES, Fuente del Cabrito, Camarena de la Sierra, Rio Camarena, 1122 m	02.vii.2016	40.16383N, -1.055242W		JM Gonzalez & J Martinez
T. (M.) cataloniensis Theowald, 1978	1♂	ES, Teruel, Camarena de la Sierra, Rio Camarena, 1216 m	02.vii.2016	40.15211N, -1.044561W		JM Gonzalez & J Martinez
T. (M.) gjipeensis sp. n.	21♂, 2♀	AL, Illias, Gjipe Gorge, 276 m	05.v.2016	40.144172N, 19.676905E		L Keresztes & LP Kolcsár
*T.* (*M.*) *siebkei* Zetterstedt, 1852	3♂	RO, Brezoi, Cozia Mts., Stanisoara Monastery, 862 m	06.vi.2000	45.302015N, 24.310468E		L Rákossy
*T.* (*M.*) *siebkei* Zetterstedt, 1852	4♂	RO, Cheia, Trascaului Mts., Cheile Turzii gorge, 444 m	11.vi.2005	46.544398N, 23.701908E		L Keresztes
*T.* (*M.*) *siebkei* Zetterstedt, 1852	1♂	IT, Balze, Monte Fumaiolo, 1159 m	18.vii.2010	43.78068N, 12.08317E		M Bálint
*T.* (*M.*) *siebkei* Zetterstedt, 1852	1♂, 1♀	RO, Pecinisca, Mehedinti Mts., Cheile Pecenisca gorge, 243 m	10.v.2013	44.850872N, 22.406507E		LP Kolcsár
*T.* (*M.*) *siebkei* Zetterstedt, 1852	2♂	RO, Capatanenii Ungureni, Fogaras Mts., Vidraru Lake surroundings, 741 m	27.v.2014	45.338783N, 24.643658E		LP Kolcsár.
*T.* (*M.*) *siebkei* Zetterstedt, 1852	1♂	IT, Balze, Monte Fumaiolo, 1159 m	18.vii.2010	43.78068N, 12.08317E		M Bálint
*T. (M.) stigmatella* Schummel, 1833	1♂	RO, Slava Chercheza, Usmenia Monastery, 132 m	03.vi.2005	44.837727N, 28.567579E		L Keresztes
*T. (M.) stigmatella* Schummel, 1833	2♂, 1♀	RO, Sasca Romana, Nera Mts., Cheile Nerei gorge, 159 m	09.v.2009	44.893238N, 21.714745E		J Csepregi
*T. (M.) stigmatella* Schummel, 1833	1♂	BG, Kulata, Struma River gorge, 84 m	04.v.2011	41.380772N, 23.36477E		LP Kolcsár
*T. (M.) stigmatella* Schummel, 1833	4♂	GR, Kavala, Batis camp area, 2 m	05.v.2011	40.934937N, 24.402779E		LP Kolcsár
*T. (M.) stigmatella* Schummel, 1833	1♂	BG, Beli Osam, Stara Planina Mts., Vila Nana, 526 m	12.vi.2012	42.856119N, 24.650439E		LP Kolcsár
*T. (M.) stigmatella* Schummel, 1833	7♂	BG, Tazha, Stara Planina Mts., Rusalka hut, 1096 m	13.vi.2012	42.688772N, 25.055851E		LP Kolcsár
*T. (M.) stigmatella* Schummel, 1833	2♂,	RO, Lunca Bradului, Mures River gorge, 576 m	14.v.2013	46.956536N, 25.103522E		LP Kolcsár
*T. (M.) stigmatella* Schummel, 1833	1♂	FYM, Jablanica Mts, Vevchani, springs, 922 m	29.iv.2018	41.239486N, 20.585719E		L Keresztes

## Taxonomy

### Description of the new species

#### Tipula (Mediotipula) gjipeensis

Taxon classificationAnimaliaDipteraDolichopodidae

Keresztes & Kolcsár sp. n.

http://zoobank.org/4CB2A315-1FF5-4DC4-84BF-202E44B9794F

Figures 2 , 3 , 4A

##### Material examined.

***Holotype***: 1 male. ***Paratypes***: 20 males, 2 females, same locality as holotype.

##### Type locality.

Albania; Vlora district, Illias, Gjipe Goerge, 267 m, 40.144172°N, 19.676905°E, 05.v. 2016, leg. L Keresztes & LP Kolcsár.

Type specimens are deposited in the Diptera Collection of the Faculty of Biology and Geology (DCFBG), University Babes-Bolyai, Cluj Napoca, Romania.

##### Diagnosis.

Males: Tergite IX–X in males with the posterior margin having a medial spinous extension with a wide base and gradually narrowed tip. Lateral corner of the posterior margin of the tergite IX–X is mostly rounded. Outer gonostylus widened gradually to tip, ending oblique at dorsal margin. The anterior end of the anterior arm of the inner gonostylus has a long beak-like elongation. The posterior arm of the inner gonostylus has in its dorsal margin a concentration of strong stout setae directed anteriorly, and the anterior corner ending with a thorn-like process. On the middle of the posterior part of the inner gonostylus a small triangular posterolateral extension is present. Females have the base of the hypogynal valves bulbous and rounded.

##### Description.

Medium sized species, Body length: holotype male 10 mm, paratype female 12 mm; wing length: holotype male 15 mm, paratype female 16 mm. Adult habitus: General colour yellowish brown. First two segments of antennae yellowish, third light brown, remainder brown. Nasus yellowish with stout black setae. Dorsal part of head, near antennae, with two whitish patches; rest of head grey-peach coloured with dark setae, except whitish yellow occipital area. Thorax -brownish grey, with four shiny brown stripes on dorsal surface. Scutellum yellowish. Wings transparent, *Mediotipula*-like, discoidal cell present. Coxae and trochanters yellowish; rest of leg segments brown. Abdomen yellowish, with dark brown patches to continuous bands on the posterior edge and lateral margin of tergites I–VII, tergite VIII entirely brown.

**Figure 2.**
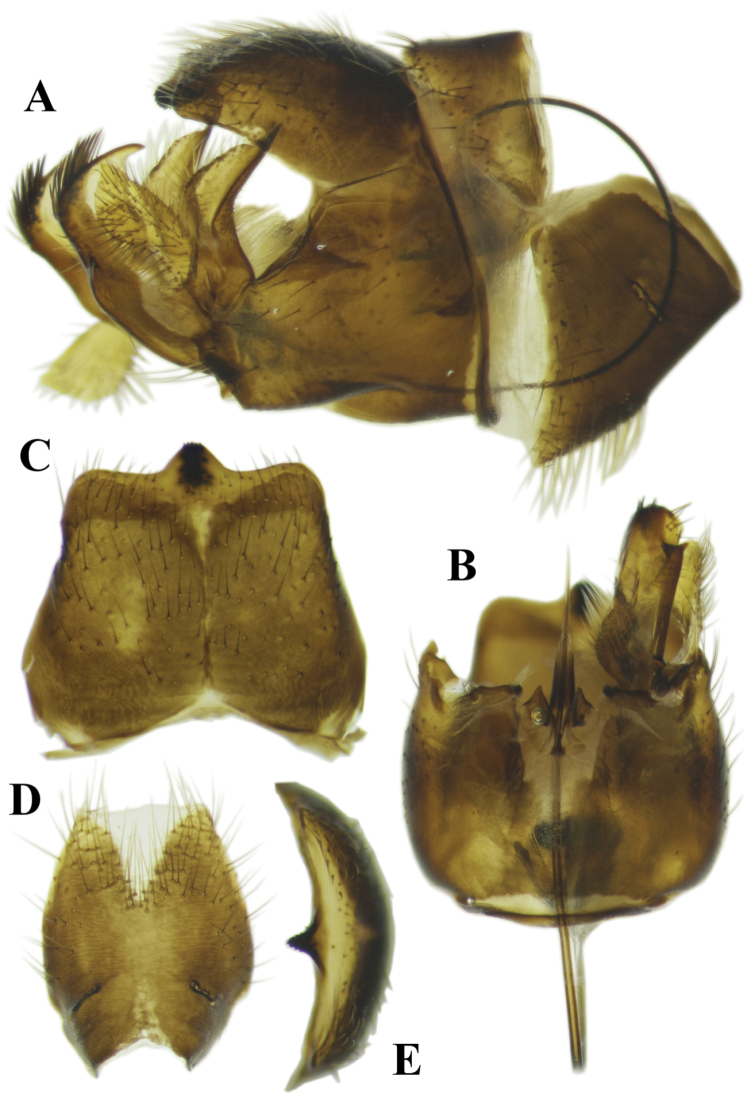
Photographs of the morphological structures of the male terminalia of the Tipula (Mediotipula) gjipeensis sp. n. **A** lateral view **B** distal view **C** tergite IX dorsal view **D** sternite VIII ventral view **E** tergite IX–X, distal view.

**Figure 3.**
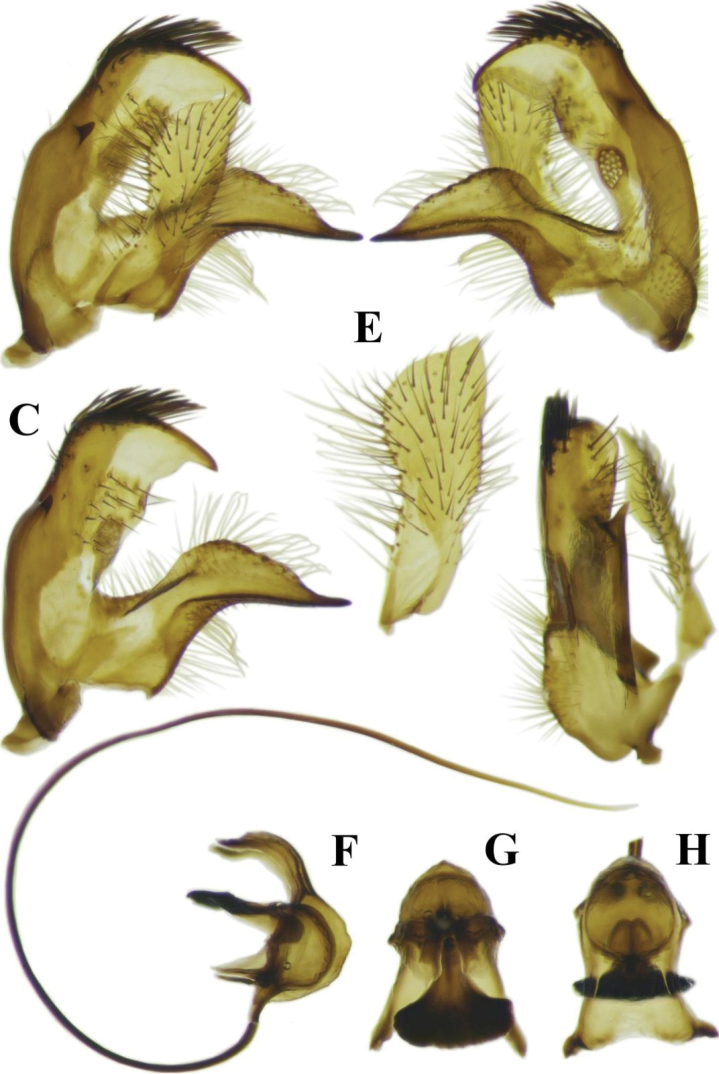
Photographs of the morphological structures of the male terminalia of the Tipula (Mediotipula) gjipeensis sp. n. **A** gonostyli outer-lateral view **B** gonostyli inner-lateral view **C** inner gonostylus outer-lateral view **D** gonostyli ventral view **E** inner gonostylus outer lateral view **F** aedeagus complex lateral view **G** sperm pump ventral view **H** sperm pump distal view.

**Male terminalia** (Figures 2, 3). Tergite IX–X with medial part bearing a narrow medial longitudinal suture with reduced whitish area close to its posterior margin (Figure 2C) and a relatively small medial spiny extension with a wide base and gradually narrowed tip on its posterior margin (Figure 2C, E). Ventral surface of posterior margin of tergite IX–X flat, lacking ventrally produced extension (Figure 2E). Lateral corner of posterior margin of tergite IX–X rounded (Figure 2C). Posterior margin of sternite 8 V-shaped, with posterior margin ending straight (Figure 2D). Gonocoxite has a laterally compressed projection on the posterodorsal corner (Figure 2A, B). Part of the gonocoxite behind the suture, on posterior part, short (Figure 2B). Interior surface of gonocoxite membranous, but with a uniformly sclerotised and relatively flat structure in the middle part. Outer gonostylus widens gradually to tip, ending obliquely at dorsal margin (Figure 3E). Lower anterior part of inner gonostylus with concentration of long setae (Figure 3A–C). Anterior end of anterior arm of inner gonostylus with a long beak -like elongation (Figure 3A–C). Posterior arm of inner gonostylus with posterior half of the dorsal margin bearing a concentration of strong thorn-like setae directed anteriorly, anterior corner ending in thorn-like process (Figure 3A–C). Posterior part of inner gonostylus with a small triangular posterolateral extension medially (Figure 3D). sperm pump with posterior apodemes fused in the horizontal plane (Figure 3F–H). Parameres small and triangular (Figure 2B).

**Figure 4.**
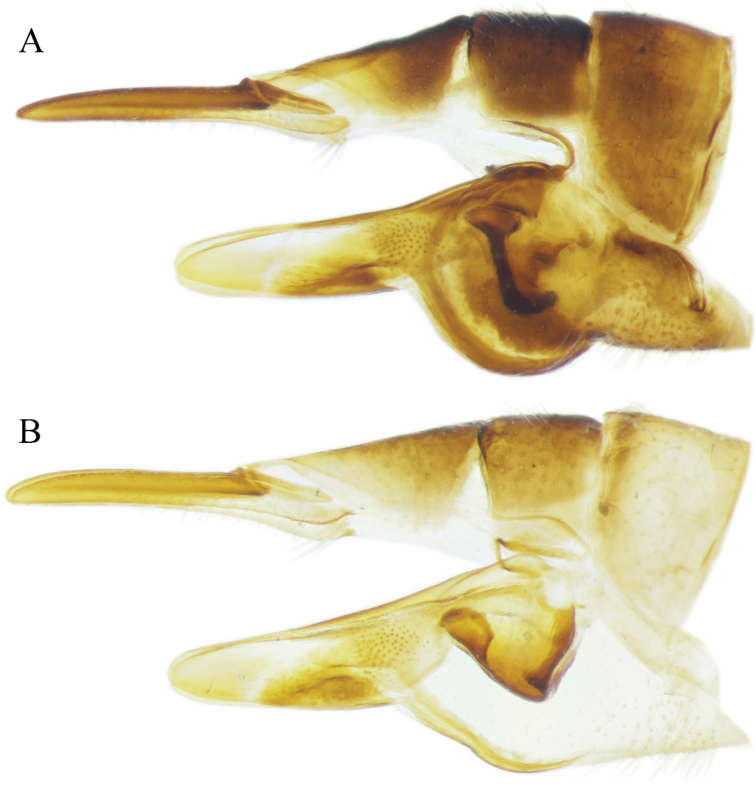
Photographs of the female terminalia, lateral view. **A**Tipula (Mediotipula) gjipeensis and **B**T. (M.) stigmatella.

**Female terminalia** (Figure 4A). Cercus slightly curved downward and terminating in a rounded apex (Figure 4A). Hypogynal valves only moderately sclerotised and fused for approx. two thirds of their length. End of membranous area of sternite VIII at base of hypogynal valves distinctly acute. Base of hypogynal valves bulbous. Ventral wall of genital chamber, near opening of gonopore is distinctly sclerotised. Vestigial sternite IX present as a slender, but well-sclerotised structure.

##### Etymology.

The species epithet *gjipeensis* translates to “from the Gjipe Gorge” and was formed by appending the Latin suffix -*ensis* to the name of the gorge where the new species was collected.

##### Ecological notes and distribution.

During our investigation in the south-western part of Albania, the new species described here was only detected in the highly-isolated humid habitat in the Gjipe Gorge, near the shore of the Adriatic Sea and very close to Gjipe Beach. The gorge is cut by a small stenotherm brook, fed by a spring ca. 2–3 km upstream. The river bed is filled with large limestone boulders and rocks, and has abundant mossy cover. The brook spring is surrounded by dense riparian vegetation, where the adults flew during daytime or were sometimes seen to rest on trees near the river. The microclimate of the river valley was roughly 10 °C cooler than in the surrounding macchia (L Keresztes pers. obs.). Specimens were collected in early May. The river was completely dry in June and no additional flying adults were collected, suggesting a short phenology between April and May (LP Kolcsár pers. obs).

## Discussion

### Systematic position and affinities of *M.gjipeensis* sp. n.

The new species belongs to the subgenus Mediotipula, a cranefly group restricted to the western Palearctic area, with the majority of taxa (eight out of twelve) having a range-restricted distribution in the Mediterranean area and the Caucasus. *Mediotipula* has a rather isolated phylogenetic position among the western Palearctic Tipulidae, sharing a combination of synapomorphies that is unique for this subgenus (De Jong 1995), and present also in *T.* (*M.*) *gjippensis*, such as the relatively small discoidal cell, the entirely fused, plate-like posterior apodemes of the sperm pump and a laterally compressed projection on the posterolateral corner of the gonocoxite in males. Additionally, females have the hypogynal valves fused for approx. one-half to two thirds of the length, in comparison with the totally separated (up to their bases) hypogynal valves in females of other *Tipula* (for more details see De Jong 1995).

**Figure 5.**
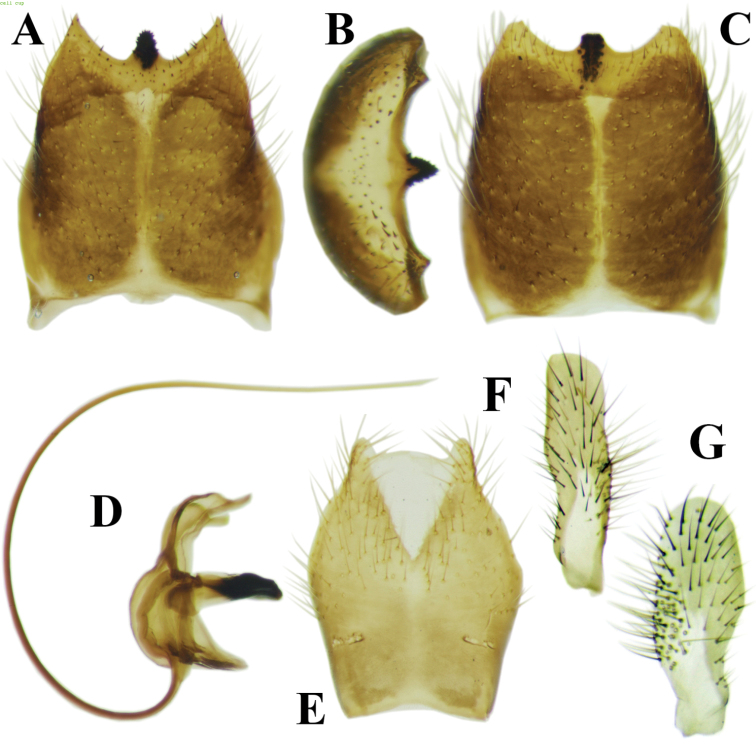
Photographs of the morphological structures of the male genital structures of individuals of Tipula (Mediotipula) stigmatella Schummel, 1833. Specimen from Kavala (Greece) **A, B, D, E, F** Specimen from Kulata (Bulgaria) **G**; Specimen from Lunca Bradului (Romania) (**C**) **A, C** tergite IX dorsal view **B** tergite IX distal view **D** aedeagus complex lateral view **E** sternite VIII ventral view **F, G** outer gonostylus, lateral view.

**Figure 6.**
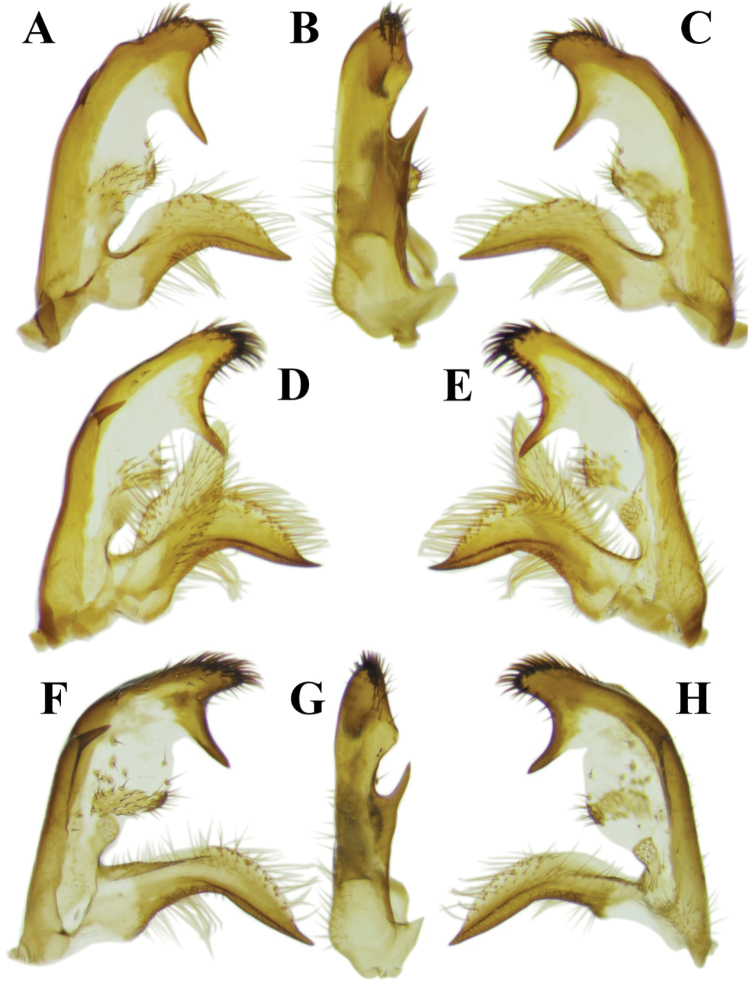
Photographs of the morphological structures of the male genital structures of individuals of Tipula (Mediotipula) stigmatella Schummel, 1833. Specimen from Kavala (Greece) **A, B, C** Specimen from Lunca Bradului (Romania) **D, E**; Specimen form Kulata (Bulgaria) (**F, G, H**) **A, F** inner gonostylus outer lateral view **B, G** inner gonostylus ventral view **C, H** inner gonostylus inner lateral view **D** gonostyli outer lateral view **E** gonostyli inner lateral view.

The new species is most similar to T. (M.) stigmatella, having the inner gonostylus of the male terminalia approx. two times as high as inner gonostyli of other species of *Mediotipula* (Figs 3D, 6B, G). However, the new species can be easily distinguished from the latter species, because in the males the posterior margin of tergite IX–X has a medial spiny extension with a wide base that gradually narrows towards the tip (Figure 2C), instead of the narrowed and rounded medial spinous extensions seen in T. (M.) stigmatella (Figure 5A, C). The medial spiny extension is relatively small (Figure 2E) in comparison with the similar structure in T. (M.) stigmatella (Figure 5B). In T. (M.) gjipeensis the lateral corner of the posterior margin of tergite IX–X is mostly rounded (Figure 2C) instead of pointed, as in T. (M.) stigmatella (Figure 5A, C) and the ventral surface of the posterior margin of tergite IX–X is flat (Figure 2E), while T. (M.) stigmatella has two triangular protuberances (Figure 5B). In T. (M.) gjipeensis males a narrow medial longitudinal suture is present on the medial part of the disk of tergite IX–X (Figure 2C), with a reduced whitish area close to the posterior margin, instead of the long light stripe in the middle part found in T. (M.) stigmatella (Figure 5A, C). In *T.* (*M.*) *gjippensis* males, the posterior margin of sternite VIII ends straight (Figure 2D), instead of having two lateral projections as in T. (M.) stigmatella (Figure 5E). The outer gonostylus widens gradually to tip, ending obliquely towards the dorsal margin (Figure 2E), while in T. (M.) stigmatella, the anterior and posterior edges of the outer gonostylus run more or less parallel, ending straight or rounded toward dorsal margin (Figure 5F, G). The posterior arm of the inner gonostylus has its dorsal margin in the posterior half with a concentration of strong thorn-like setae directed anteriorly, anterior part bare, ending with a thorn-like process (Figure 3A–C). while in T. (M.) stigmatella a concentration of strong setae covers the entire dorsal margin of the posterior arm of the inner gonostylus, with the anterior corner rounded, but a conspicuous subterminal acute process directed ventrally is present (Figure 6A, C–E, F, H). In *M.gjipeensis*, the posterior part of the inner gonostylus has a small triangular posterolateral extension present in the middle (Figure 3D), differing from the long thorn-like process displayed in T. (M.) stigmatella (Figure 6B, G) and *T.* (*M.*) *sarajevensis*, while in all other members of *Mediotipula* such processes are absent. Parameres in the new species are small and triangular (Figure 2B), similar to T. (M.) stigmatella, but not lobe-like and spiny, as in *T.* (*M.*) *siebkei* (De Jong 1995). The females of the new species are clearly distinct from all other *Mediotipula* in having a bulbous base of the hypogynal valve (Figure 4A), rather than the gradually narrowed base found in all other species except T. (M.) stigmatella. However, T. (M.) gjipeensis is also distinct from T. (M.) stigmatella in having the bulbous base of the hypogynal valve rounded, instead of angular (Figure 4B).

### Morphological variability of *M.stigmatella* in the Carpathian-Balkan area

During our investigation, morphological differences were detected between allopatric populations of T. (M.) stigmatella in the Carpathian-Balkan area. Most important divergences were detected in the shape of the posterior margin of the tergite IX–X between populations from the Carpathians (Figure 5C) and the Balkans (Figure 5A), but no important differences in the general shape of the outer and inner gonostylus were found between populations from Lunca Bradului (the Carpathians) (Figure 6D, E) and Kavala (Greece) (Figs 5F, 6A–C). However, a series of important structures were detected between more proximal populations from Kavala (Greece) together with individuals from Vevchani (FY of Macedonia) and Kulata (Bulgaria), mostly on the width of the outer gonostylus (Figure 5F, G), and on the apex of the posterior arm of the inner gonostylus (Figure 6A–C, F–H). The triangular-like apex of the posterior side of the posterior arm of the inner gonostylus of the specimens from Kavala (Greece) and Vevchani (FY of Macedonia) is shorter, with a broader base (Figure 6B), while in specimens collected from Kulata (Bulgaria) such similar structure is longer, with a narrowed base (Figure 6G). Without having a clear geographic pattern of the detected morphological divergences in the studied population, and with only a limited number of individuals, more data is required, covering the whole of the species distribution area, in order to evaluate the taxonomic importance of the detected differences.

### The eastern Mediterranean area as a diversity hotspot

Current systematic and phylogeographical studies of the Mediterranean terrestrial fauna (Oosterbroek and Arntzen 1992, De Jong 1998, Sloan et al. 2014) reveals complex origins and evolutionary histories of temperate taxa in relation with the complex paleoecological history of the area. High levels of species diversity are related to ancient events (Paleocene-Miocene) and also to more recent events (Pliocene-Pleistocene) followed by active speciation and even explosive radiations, leading finally to the emergence of the so-called “Mediterranean Sanctuary” of diversity, the second largest hotspot in the world and the largest of the world’s five Mediterranean-climatic regions (Grabowski et al. 2017, Vargas et al. 2018). Albania belongs to the Eastern Mediterranean chorotype of the fauna, as proposed by Taglianti et al. (1999) including the NE-Mediterranean, Palestino-Cyprioto-Taurian, Palestino-Taurian, and Aegean distribution patterns of different animal species.

The eastern Mediterranean area, where the new member of the subgenus Mediotipula was collected, is recognised as an important centre of endemism, but no range-restricted *Mediotipula* species were detected in the area to date. *Tipula* (*M.*) *gjipeensis* sp. n. was identified by us only in a small, humid limestone gorge in Albania. We hypothesised a restricted distribution of the species, most probably as a result of the presently isolated distribution of humid ecosystems in the Mediterranean area. Several aquatic and semi-aquatic organisms have similarly restricted or fragmented distributions in the Mediterranean area, which most probably followed the general decline of humid ecosystems during the late Cainozoic and simultaneous retreat in highly-fragmented local populations (sometimes highly-divergent evolutionary significant units, or in many cases distinct species) (Fernandez-Mazuecos et al. 2014, Godunko et al. 2017, Neubauer et al. 2016).

However, a number of case studies on plant species (Allegrucci et al. 2017, Crowl et al. 2015) hypothesise an evolutionary process in lineages that are adapted to pre-Mediterranean (pre-Pliocene) conditions in relatively small, xeric areas. Later, they became strongly competitive and expanded as the Mediterranean climate became dominant (Pliocene-Quaternary) across the Mediterranean Basin, and most probably also began to penetrate into the northern parts of Europe. Similar patterns were detected in the case of the *Lacertaviridis* complex (Cassel-Lundhagen 2010), with leading edge populations that colonised large parts of Europe, but several fringe populations became genetically divergent or morphologically different in highly isolated populations in the Balkan area. This pattern is also most likely true for the sibling pairs *T.* (*M.*) *stigmatella / T.* (*M.*) *gjipeensis*, with a larger distribution in the south-central part of Europe to Caucasus, but with morphologically deeply divergent T. (M.) gjipeensis from Albania, and less, but notable morphological differences between populations of T. (M.) stigmatella in the Balkan area, identified for example in Kavala (Greece) and Kulata (Bulgaria) (Figure 6B, G). The presence of morphologically divergent allopatric populations of T. (M.) stigmatella (possibly cryptic species) is likely a result of more recent isolation, followed by repeated dispersions and area fragmentations during the Pleistocene climate change. This needs to be subjected to a more comprehensive analyses of the species over its whole range.

### Faunistic and ecological notes

Albania is located in the western part of the Balkan Peninsula and is one of the most important Mediterranean biodiversity hotspots (Caković et al. 2018, Szabolcs et al. 2017), but is still considered to be the last “terra incognita” for European biodiversity. A series of recent studies, which focused on different groups of plants and animals, brings major contributions to the diversity and distribution of a number of organisms from Albania (Barina et al. 2013, Graf et al. 2018, Kaltsas et al. 2008, Stevanović et al. 2007). The description of *T.Mediotipulagjipeensis* sp. n. is an important addition to the faunal list of the Albanian Tipulidae and highlights the importance of similarly isolated pristine ecosystems to local and regional diversity of the Mediterranean Area.

Ecological demands of the majority of *Mediotipula* species are largely overlooked because of a high number of undescribed larvae. Based on adult distributions, species inhabiting open woods and hedges exposed to the sun seem to have generally larger distributions (e.g., *T.* (*M.*) *stigmatella*, *T.* (*M.*) *sarajevensis* etc.) in comparison with species that are restricted to steep river valleys (e.g., *T.* (*M.*) *brolemani*, *T.* (*M.*) *mikiana*, *T.* (*M.*) *gjipeensis* sp. n.) (Oosterbroek 2018). Such humid and sheltered “ecological islands” set in larger xeric areas could act as refuges for a number of range-restricted or endemic species. For example, within the steep Gjipe Gorge (only a few kilometres in length), other than *T.* (*M.*) *gjipeensis*, the Mediterranean cranefly species Dolichopeza (D.) furiscipes Bergroth, 1889 was also detected (L Keresztes unpublished data), having a fragmented distribution in similar habitats in the southern part of continental Europe, but also on some Mediterranean islands (Mederos Lopez 2012, Oosterbroek 2018). The mayfly *Electrogenahellenica* Zurwerra & Tomka, 1986 has an even more restricted distribution in the Mediterranean area which is limited to western Greece, along rivulets in steep valleys at 300 m a.s.l. (Bauernfeind and Soldan 2012), but which was also collected by the first author of this paper from the Gjipe Gorge (L Keresztes unpublished data). Similar ecosystems in the Mediterranean area are rare and isolated but they are seriously affected by human activity (limestone and rock quarries, water extraction, habitat deterioration, discharge of sewage effluents, etc.) enforced by ongoing global climate change and therefore needing special attention and perhaps protection.

## Supplementary Material

XML Treatment for Tipula (Mediotipula) gjipeensis
